# Indications and outcome of intravitreal bevacizumab injection in a Community Eye Hospital, Nepal

**DOI:** 10.1186/s12886-026-05039-6

**Published:** 2026-06-18

**Authors:** Sunil Thakali, Mohini Shrestha, Aleena Gauchan, Hom Bahadur Gurung

**Affiliations:** 1 Hetauda Community Eye Hospital, Hetauda, Nepal; 2 Tilganga Institute of Ophthalmology, Kathmandu, Nepal

**Keywords:** Diabetic retinopathy, Neovascular aged-related macular degeneration, Central retinal vein occlusion, Branch retinal vein occlusion, Intravitreal bevacizumab injection, Central macular thickness, Visual acuity

## Abstract

**Background:**

This study aimed to evaluate the indications and real-world treatment outcomes of intravitreal bevacizumab in a resource-limited community eye hospital setting.

**Methods:**

This retrospective study included patients who received IVB injection at Hetauda Community Eye Hospital between January 2019 and December 2022. Inclusion criteria:$\:\ge\:$1 IVB injection; retinal diagnosis confirmed clinically and by Optical Coherence Tomography (OCT) where available. Exclusion criteria: missing baseline Visual Acuity (VA) or OCT,$\:<1$ month follow-up. Median visual acuity (VA) and central macular thickness (CMT) changes were compared using Wilcoxon signed‑rank test.

**Results:**

A total of 418 injections were administered to 247 patients (260 eyes). After excluding those lost to follow‑up, 221 patients (234 eyes) were included in the analysis. Median baseline CMT was 366 μm and improved to 236 μm (*p* < 0.001). Most patients received one injection (57%) followed by two injections (30%). The median baseline VA was 0.78 log MAR and remained 0.78 log MAR overall (*p* = 0.108), but subgroup improvements were significant in DME, nAMD, BRVO, and central retinal vein occlusion (CRVO).

**Conclusion:**

IVB resulted in significant anatomical and functional improvements in patients with retinal diseases. The findings reflect real‑world outcomes in a community‑level eye hospital, where cost barriers limit repeated injections. The limitations of this study include its retrospective design, variable follow‑up, absence of control group, and limited numbers of injections.

## Introduction

Bevacizumab is a monoclonal antibody against vascular endothelial growth factor A(VEGF-A) that was approved by the FDA in 2004 for metastatic colorectal cancer; however, its ophthalmic use is off-label. Off-label use in ocular diseases was pioneered by Rosenfeld and has since gained widespread acceptance due to its demonstrated safety and efficacy in comparison to ranibizumab [1–3].Cost-effectiveness analyses using long-term Markov models have shown that bevacizumab provides greater economic value than ranibizumab and other treatment alternatives, assuming comparable safety and effectiveness [4]. Today, intravitreal bevacizumab(IVB) injections are common in many conditions relating to the retina: proliferative diabetic retinopathy, diabetic macular edema (DME), choroidal neovascularization (CNV) secondary to pathologic myopia or AMD, retinopathy of prematurity, macular edema due to branch and central retinal vein occlusion (BRVO and CRVO), iris neovascularization, neovascular glaucoma(NVG) and pseudophakic macular edema [5, 6]. Retinal diseases are among the leading causes of visual impairments worldwide. In Nepal, besides cataract, they are at the top of the list as the second most common cause of blindness [7, 8]. IVB injection has demonstrated benefits by lowering VEGF levels, accelerating the resolution of hemorrhage, and decreasing the need for invasive procedures in diabetic retinopathy and in retinal vein occlusion. It may also be used as a potentially active alternative treatment for other diseases like central serous chorioretinopathy [9–14]. 

This study highlights the role of a comprehensive ophthalmologist in managing medical retinal diseases within a community hospital setting, an area often underrepresented in the existing literature that tends to focus on tertiary centers or retina specialists. Conducted in Makwanpur district, the study emphasizes the accessibility of medical retina services to the local community, reducing the financial burden and inconvenience for patients, especially those with limited resources, by eliminating the need to travel to tertiary centers.

Furthermore, despite the widespread use of intravitreal bevacizumab for various retinal conditions, there is limited data from Nepal regarding its real-world outcomes [15–18]. Given the socioeconomic challenges faced by patients in these regions, this study aimed to provide evidence-based recommendations to improve patient care by evaluating the effectiveness of this treatment. In addition, there have been no similar study in this part of Nepal.

Bevacizumab offers greater economic value than ranibizumab and other available treatment alternatives, assuming comparable safety and effectiveness [19]. Although intravitreal bevacizumab (IVB) has been widely studied in Low- and Middle-Income Countries (LMICs), including Nepal [20–22] most evidence comes from tertiary eye centers with stronger resources and follow-up systems, leaving limited real-world data from Nepal’s community-level hospitals despite its broad use across multiple retinal conditions [14–17]. Our study addresses this gap by evaluating IVB outcomes in a real-world community hospital setting where patients often encounter significant socioeconomic barriers, including limited affordability, travel difficulties, and irregular follow-up. Additionally, we assess IVB use across a broader spectrum of retinal diseases, including DME, nAMD, RVO, PCME, CSR, hypertensive retinopathy, and circinate retinopathy, providing practical insights into treatment effectiveness and challenges in resource-limited settings. To our knowledge, no similar study has been conducted in this region of Nepal.

This study aimed to evaluate anatomical and functional outcomes of IVB injections for various retinal diseases at a community eye hospital in Nepal.

## Material and method

### Study design

This was a retrospective study of patients with retinal diseases admitted to Hetauda Community Eye Hospital who received Intravitreal Bevacizumab Injections from 2019 to 2022. Data collection started on November 5, 2023, after obtaining approval from the Institutional Review Board, Tilganga Institute of Ophthalmology. Data extracted at every visit included visual acuity on the Snellen chart, which was transformed into log MAR; measurement of intraocular pressure by Goldman applanation tonometry; fundus examination; and central macular thickness measurement of the retina with SD-OCT using the 3D OCT-1 Maestro by Topcon. Data were retrieved from electronic medical records. During the study period, intravitreal bevacizumab was the only anti-VEGF agent used in our setting, as alternative agents such as ranibizumab and aflibercept were not utilized due to limited availability and affordability.

### Study outcomes

The primary outcome measures were changes in visual acuity (log MAR) and central macular thickness (CMT) between baseline and the final available follow-up visit. Final visual acuity and CMT values were defined as the last recorded measurements for each eye during follow-up.

### Inclusion criteria

- Patients who received intravitreal bevacizumab between January 2019 and December 2022.
- Received at least one IVB injection.
- Minimum follow-up duration of one month after the first injection.
- Definitive diagnosis of one of the following retinal conditions:
  - Diabetic macular edema (DME).
  - Neovascular age-related macular degeneration (nAMD).
  - Macular edema secondary to BRVO or CRVO.
  - Pseudophakic cystoid macular edema (PCME).
  - Central serous chorioretinopathy (CSR).
  - Retinal vasculitis.
  - Hypertensive retinopathy with macular edema.

### Exclusion criteria

- Patients who lost follow-up before the first post-injection evaluation.
- Missing baseline OCT required for CMT analysis.
- Significant media opacity (e.g., dense cataract, vitreous hemorrhage) preventing fundus or OCT assessment.

### Case definitions

#### DME

Retinal thickening involving the macula with intraretinal cysts or subretinal fluid on OCT in a patient with diabetes.

#### nAMD

Presence of choroidal neovascularization with associated subretinal/intraretinal fluid or hemorrhage on OCT or clinical exam.

#### **BRVO/CRVO** macular edema

Macular thickening with intraretinal fluid in an eye with a clinical diagnosis of branch or central retinal vein occlusion.

#### PCME

Presence of cystoid macular edema on OCT at least 4 weeks after an uneventful cataract surgery.

#### CSR

Serous neurosensory retinal detachment confirmed on OCT without identifiable causes.

#### Retinal vasculitis

Signs of vascular sheathing or inflammation with corresponding macular edema on OCT.

#### Hypertensive retinopathy with cystoid macular edema (CME)

Macular thickening associated with hypertensive retinal changes.

### Injection technique

All injections were performed in the operating room. Bevacizumab (Genentech Inc., San Francisco, CA, USA) 1.25 mg/0.05 mL, was aspirated into a 1 mL syringe with a 20/23 G needle from the vial (100 mg/4mL). Each 1 ml syringe was capped with a 30 G needle to ensure no air was present in the syringe. The surgeon and patient wore sterile gowns, caps, and masks. Eyelashes, eyelids, and caruncle were swabbed with Betadine 10%. Lid speculum and drape exposed to the surgical area. They were injected at the supertemporal using a 30-G needle at 3.5- or 4-mm posterior to the limbus in pseudophakia or phakic respectively with the patients looking down. Finally, ciprofloxacin ointment patching was performed on the eye for two hours’ post-injection and the patient was treated with ciprofloxacin eye drops four times daily for one week.

Written post-operative instructions were given to the patients, and they were informed about the warning signs like ocular pain, decreased vision, and lid edema. Follow up was performed after 1 week, 1 month, and subsequently every month. Reinjection was done after a minimum of four weeks from the date of primary injection. These are routine practices at Hetauda Community Eye Hospital. Treatment decisions and follow-up intervals were influenced by real-world factors such as patient affordability, accessibility, and compliance, resulting in variability in injection frequency and follow-up patterns.

### Data analysis

Data entry, cleaning, and coding were performed in Microsoft Excel and analyzed using IBM SPSS V.26. The Shapiro-Wilk test assessed the normality of the differences in pre- and post-vision and CMT data. Owing to non-normal distributions, the Wilcoxon signed-rank test was used, with a p-value < 0.05 considered significant.

CMT improvement was calculated as baseline CMT (cmt0) minus final CMT (cmtF), yielding:

- Positive value: improvement
- Zero: no change
- Negative value: worsening.

Vision improvement was calculated similarly as the baseline log MAR vision (va0) minus the final log MAR vision (vaF).

Analyses were performed at the eye level, as clinical assessments and treatment outcomes were recorded per eye in this study.

## Results

This study included a review of 418 injections administered to 247 patients (260 eyes). After excluding 26 patients lost to follow-up, 234 eyes of 221 patients were included in the study. Of the latter, 29 had no OCT, as only visual acuity was analyzed. The mean age of the 221 patients was 64.4 ± 12 years, ranging from 20 to 88 years. The sex composition included 124 males (56.1%) and 97 females (43.9%). Most of the IVB injections were administered to patients from Makwanpur district, followed by Bara district. Diabetic macular edema, neovascular age-related macular degeneration, branch retinal vein occlusion, and central retinal vein occlusion were common indications for IVB injections. In detail, demographic features and indications are summarized in Tables 1 and 2.

**Table 1:** Baseline demographic characteristics

Characteristics	Number (%)
**Laterality**	
Bilateral	13 (5.9)
Unilateral	208 (94.1)
**Ethnicity**	
Hill Brahman	87 (39.4)
Hill/Mountain Janajati	57 (25.8)
Hill Chhetri	35 (15.8)
Newar	22 (10.0)
Hill Dalit	14 (6.3)
Tarai/Madhesi other castes	5 (2.3)
Others	1 (0.5)
**Address**	
Makwanpur	209 (94.6)
Bara	5 (2.3)
Sarlahi	3 (1.4)
Rautahat	3 (1.4)
Chitwan	1 (0.5)

**Table 2:** Indication of intravitreal bevacizumab

Diagnosis	Number (%)
Diabetic Macular Edema (DME)	84 (35.9)
nAMD	68 (29.1)
BRVO	57 (24.4)
CRVO	9 (3.8)
Pseudophakic Cystoid Macular Edema	6 (2.6)
CSR	5 (2.1)
Circinate Retinopathy	2 (0.9)
Retinal Vasculitis with Vitreous bleed	2 (0.9)
Hypertensive Retinopathy with CME	1 (0.4)
Total	234 (100.0)

nAMD: Neovascular age-related macular degeneration, BRVO: Branch retinal vein occlusion, CRVO: Central retinal vein occlusion, CSR: Central serous chorioretinopathy, CME: Cystoid macular edema

The median baseline CMT was 366 μm, ranging from 72 to 824 μm, and the median baseline VA was 0.78 log MAR, ranging from 0 to 3.7 log MAR. The median CMT significantly improved to 236 μm (range 74–877 μm) at the end of follow-up (*p* < 0.001), whereas the median best-corrected VA remained 0.78 log MAR (*p* = 0.108). Statistically significant improvement in CMT was observed in BRVO, while visual acuity showed statistically significant improvement in BRVO, nAMD, and DME. The mean change in BCVA from baseline was 0.06 log MAR for DME, -0.11 log MAR for nAMD, 0.16 log MAR for BRVO, 0.25 log MAR for CRVO, 0.09 log MAR for PCME, and 0.22 log MAR for CSR.For other diagnostic subgroups with small sample sizes, only descriptive changes are reported, and no subgroup-specific conclusions regarding treatment effectiveness can be drawn. The detailed changes in CMT and VA across different pathologies are presented in Tables 3 and 4.

**Table 3:** Central macula thickness (CMT) at baseline and final follow-up

Diagnosis	*n*	Baseline CMT (µm)	Final CMT (µm)	Difference in CMT	*p-value
		Median (Range: Min to Max)	Median (Range: Min to Max)	Median (Range: Min to Max)	
DME	69	356 (179 to 646)	273 (104 to 639)	45 (-308 to 453)	0.002
nAMD	60	306.5 (72 to 586)	232 (74 to 877)	45 (-535 to 362)	0.088
BRVO	54	480 (124 to 824)	211.5 (104 to 504)	197.5 (-132 to 623)	< 0.001
CRVO	9	550 (264 to 718)	204 (138 to 538)	361 (-75 to 580)	0.021
CSR	5	210 (118 to 661)	138 (107 to 233)	102 (-18 to 428)	0.138
Pseudophakic Cystoid Macular Edema	5	496 (313 to 558)	299 (224 to 549)	34 (-4 to 334)	0.463
Total	205	366 (72 to 824)	236 (74 to 877)	71 (-535 to 623)	< 0.001

*p value generated using the Wilcoxon Signed Rank test

DME: Diabetic macular edema, nAMD: Neovascular age-related macular degeneration, BRVO: Branch retinal vein occlusion, CRVO: Central retinal vein occlusion, CSR: Central serous chorioretinopathy, µm: micrometer, n: number

**Table 4:** Visual acuity (VA) at baseline and final follow-up (log Mar)

Diagnosis	*n*	Baseline VA	Final VA	Difference in VA	*p-value
		Median (Range: Min to Max)	Median (Range: Min to Max)	Median (Range: Min to Max)	
DME	84	0.78 (0 to 3.7)	0.6 (0 to 3.7)	0 (-3.7 to 3.22)	< 0.001
nAMD	68	1 (0.18 to 2.9)	1 (0.18 to 2.9)	0 (-2.42 to 2.42)	0.001
BRVO	57	0.78 (0 to 2.9)	0.6 (0 to 3.7)	0.18 (-0.8 to 1.48)	< 0.001
CRVO	9	1 (0.78 to 3.7)	1 (0.3 to 3.7)	0.22 (-0.3 to 1)	0.021
Pseudophakic Cystoid Macular Edema	6	1 (0.6 to 1.78)	0.78 (0.48 to 1.78)	0.06 (-0.78 to 1)	0.08
CSR	5	0.48 (0 to 1.78)	0.48 (0 to 1)	0 (-0.3 to 1.3)	0.138
Total	234	0.78 (0 to 3.7)	0.78 (0 to 3.7)	0 (-3.7 to 3.7)	0.108

*p value generated using the Wilcoxon Signed Rank test

DME: Diabetic macular edema, nAMD: Neovascular age-related macular degeneration, BRVO: Branch retinal vein occlusion, CRVO: Central retinal vein occlusion, CSR: Central serous chorioretinopathy, Log Mar: logarithm of the minimum angle of resolution

The number of IVB injections for various retinal diseases is presented in Table 5. DME has 84 injections in total, which makes up 35.9% of all injections. nAMD follows with 68 injections, which are 29.1%. BRVO is the third most prevalent with 57 injections, or 24.4%. The number of patients decreases significantly with the increasing number of injections.

**Table 5:** The number of IVB injections to various retinal diseases

Diagnosis	Number of IVB Injection						
	One	Two	Three	Four	Five	Eight	Total
	*n* (%)	*n* (%)	*n* (%)	*n* (%)	*n* (%)	*n* (%)	*n* (%)
DME	58 (43.6)	19 (27.1)	3 (20)	2 (16.7)	1 (50)	1 (50)	84 (35.9)
n AMD	32 (24.1)	24 (34.3)	8 (53.3)	3 (25)	0 (0)	1 (50)	68 (29.1)
BRVO	29 (21.8)	20 (28.6)	2 (13.3)	5 (41.7)	1 (50)	0 (0)	57 (24.4)
CRVO	3 (2.3)	3 (4.3)	1 (6.7)	2 (16.7)	0 (0)	0 (0)	9 (3.8)
Pseudophakic Macular Edema	3 (2.3)	2 (2.9)	1 (6.7)	0 (0)	0 (0)	0 (0)	6 (2.6)
CSR	4 (3)	1 (1.4)	0 (0)	0 (0)	0 (0)	0 (0)	5 (2.1)
Retinal Vasculitis with Vitreous hemorrhage	2 (1.5)	0 (0)	0 (0)	0 (0)	0 (0)	0 (0)	2 (0.9)
Circinate Retinopathy	1 (0.8)	1 (1.4)	0 (0)	0 (0)	0 (0)	0 (0)	2 (0.9)
Hypertensive Retinopathy with CME	1 (0.8)	0 (0)	0 (0)	0 (0)	0 (0)	0 (0)	1 (0.4)
Total	133 (100)	70 (100)	15 (100)	12 (100)	2 (100)	2 (100)	234 (100)

DME: Diabetic macular edema, nAMD: Neovascular age-related macular degeneration, BRVO: Branch retinal vein occlusion, CRVO: Central retinal vein occlusion, CSR: Central serous chorioretinopathy, n: number

## Discussion

The mean age of 221 patients in our study was 64.4 years ± 12 years, ranging from 20 to 88 years, similar to a Kathmandu-based study where the mean age was 59.62 years (range 19–91) [15]. Diabetic macular edema was the most common indication for intravitreal bevacizumab(IVB) injections, reflecting the high prevalence of this disease and the visual impairment associated with diabetes mellitus.

Diabetic macular edema was the most common indication for intravitreal bevacizumab injections, reflecting the high prevalence of diabetes mellitus and its associated visual impairment. Pathologies such as macular edema, vitreous hemorrhage, and tractional retinal detachment remain major causes of blindness among the working population worldwide [23, 24]. The predominance of DME as the primary indication aligns with studies conducted in Nepal, Uganda, and Pakistan, highlighting its widespread impact [15, 16, 25, 26].

In our study, nAMD was the second most common indication for IVB injections, unlike others where it usually occupies the third position after DR and RVO [15, 16, 26, 27]. The increased life expectancy of the Nepali population in the current situation has increased the risk of nAMD and the cost-effectiveness of IVB injection has driven its widespread use, despite the lack of formal licensing for ocular conditions [27–29].

RVO, including both BRVO and CRVO, was the third most common indication. Some studies have reported higher IVB usage for RVO compared to nAMD, highlighting regional differences in treatment patterns and disease prevalence [15, 16, 25, 26, 30].

Pseudophakic cystoid macular edema (PCME) was the fourth most common indication for IVB injection in our study. Previous studies have reported improvements in macular thickness and visual acuity following intravitreal bevacizumab treatment in patients with PCME. 6)Central serous chorioretinopathy (CSR) was the fifth most common indication, consistent with reports from Nepal and Pakistan, where CSR represented a relatively uncommon indication for IVB use [15, 26].

A small number of eyes with circinate retinopathy, hypertensive retinopathy with macular edema, and retinal vasculitis with vitreous hemorrhage also received IVB injections. Given the limited number of cases, no conclusions regarding treatment effectiveness can be drawn from our data. However, previous studies have suggested that intravitreal bevacizumab may help reduce macular edema and improve visual outcomes in selected patients with circinate retinopathy and hypertensive retinopathy, and may have a role in managing retinal vasculitis-associated complications [31–34]. 

The effect of IVB injection was further examined by disease diagnosis, and the results showed a modest change in CMT, there was significant improvement of VA by IVB among patients suffering from diabetic macular edema, which is corroborated by findings from a similar study [25]. The good effect of IVB in DME, for both CMT and VA, is reported in many previous studies [17, 18, 35, 36].

There was an improvement in CMT and VA for patients with BRVO, consistent with reports from other studies [30, 37, 38]. Our study showed that CMT and VA in patients with CRVO did not change significantly. This contrasts with studies that have shown significant improvements after IVB injection, being comparable to the improvements achieved with intravitreal injections of Ranibizumab [13]. The poor visual outcome in our study may have been influenced by the undetected ischemic CRVO and the variable timing of Bevacizumab injections [39, 40]. Visual and anatomic outcomes in our setting are inferior to those in other trials and studies, which may be due to the lack of implementation of optimal diagnostic and retreatment criteria.

In comparison, patients with RVO showed less VA improvement with IVB injection than those with nAMD [30]. The greater VA gain and CMT reduction in nAMD justify the off-label use of bevacizumab as a highly cost-effective intervention for this condition [28].

While in our study, VA and CMT did not show any significant change with the use of IVB for Pseudophakic cystoid macular edema, the Pan-American Collaborative Retina Study Group did report significant improvements [6]. Similarly, IVB in CSR showed no significant changes in our study, but it remains a promising treatment that requires further investigation [14].

Our study evaluated the effect of IVB injection for a variety of retinal diseases one month after injection. We found that, in general, IVB injection led to anatomical and functional improvements. Unlike most studies, which focused on the condition, such as diabetic macular edema or retinal vein occlusion, our research considered a wider spectrum of retinal pathologies.

However, most patients received only one or two injections (57% and 30% respectively), which is fewer than the number of injections recommended by standard protocols for these conditions. These findings should therefore be interpreted within the context of real-world practice, where treatment frequency is often limited by affordability, accessibility, and patient adherence in a community hospital setting. This results in outcomes lower than those reported in large clinical trials. Despite the lower number of injections, meaningful anatomical and functional improvements were observed, reflecting real-world outcomes in a resource-limited context. Differences in follow-up duration and limited retreatment reduce comparability with international studies. Nonetheless, the findings reflect genuine community-level challenges in Nepal and highlight the need for subsidized anti-VEGF therapy.

### Strength

This study provides several unique contributions to the existing evidence on intravitreal bevacizumab in low-resource settings. First, it represents the earliest analysis from Makwanpur district and one of the few studies conducted in a community eye hospital rather than a tertiary center, thereby reflecting the true real-world burden of retinal diseases in rural and semi-urban areas of Nepal. Second, it demonstrates the feasibility of delivering medical retina services by comprehensive ophthalmologists, an underreported but critical strategy for improving access to LMICs. Third, the study includes a wide range of retinal diagnoses commonly encountered in community practice, offering a holistic view of IVB use in diverse retinal pathologies.

### Limitation

This study has several limitations. First, its retrospective design restricts the ability to control confounding variables and limits causal interpretation. Second, the duration of follow-up was short and variable across patients, which may have affected the measurement of long-term anatomical and functional outcomes. The study did not adjust for number of injections, baseline disease severity, duration of follow-up, ischemic status, or inter-eye correlation in patients contributing both eyes. These factors may have influenced outcomes and should be considered when interpreting the results. Third, a high proportion of eyes received only one intravitreal injection, largely due to socioeconomic and accessibility constraints, which likely influenced the overall treatment response. Fourth, the absence of a control group prevented direct comparison with alternative treatment strategies or natural disease progression. Finally, OCT data were unavailable for 29 eyes, reducing the completeness of anatomical analysis for those cases.

## Conclusion

The prevalence of retinal diseases is increasing, creating a growing need for accessible treatment options such as intravitreal bevacizumab (IVB). In this study, IVB use was associated with improvements in visual acuity and reduction in central macular thickness across various retinal conditions, with diabetic macular edema, neovascular age-related macular degeneration, and retinal vein occlusions being the most common indications. However, these findings reflect real-world outcomes in a resource-limited setting, where treatment frequency was often suboptimal due to socioeconomic and accessibility constraints. Therefore, our results support existing evidence on the utility of IVB while highlighting the challenges of achieving optimal treatment outcomes in community-based practice.

## Data Availability

Data is provided within the manuscript or supplementary information files. However, the raw data underlying these findings cannot be shared publicly due to patient consent constraints. De-identified data can be made available upon reasonable request. Parties interested in accessing these data should contact the corresponding author, who will request permission from Hetauda Community Eye Hospital before providing access.

## Abbreviations

DME: Diabetic macular edema
nAMD: Neovascular age-related macular degeneration
BRVO: Branch retinal vein occlusion
CRVO: Central retinal vein occlusion
CSR: Central serous chorioretinopathy
CME: Cystoid macular edema
IVB: Intravitreal Bevacizumab
VA: Visual acuity in log MAR
log MAR: Logarithm of the minimum angle of resolution
CMT: Central macular thickness
